# Clastogenic Effects of Glyphosate in Bone Marrow Cells of Swiss Albino Mice

**DOI:** 10.1155/2009/308985

**Published:** 2008-12-15

**Authors:** Sahdeo Prasad, Smita Srivastava, Madhulika Singh, Yogeshwer Shukla

**Affiliations:** Proteomics Laboratory, Indian Institute of Toxicology Research, Mahatma Gandhi Marg, Lucknow 226001, India

## Abstract

Glyphosate (N-(phosphonomethyl) glycine, C_3_H_8_NO_5_P), a herbicide, used to control unwanted annual and perennial plants all over the world. Nevertheless, occupational and environmental exposure to pesticides can pose a threat to nontarget species including human beings. Therefore, in the present study, genotoxic effects of the herbicide glyphosate were analyzed by measuring chromosomal aberrations (CAs) and micronuclei (MN) in bone marrow cells of Swiss albino mice. A single dose of glyphosate was given intraperitoneally (*i.p*) to the animals at a concentration of 25 and 50 mg/kg b.wt. Animals of positive control group were injected *i.p*. benzo(a)pyrene (100 mg/kg b.wt., once only), whereas, animals of control (vehicle) group were injected *i.p*. dimethyl sulfoxide (0.2 mL). Animals from all the groups were sacrificed at sampling times of 24, 48, and 72 hours and their bone marrow was analyzed for cytogenetic and chromosomal damage. Glyphosate treatment significantly increases CAs and MN induction at both treatments and time compared with the vehicle control (*P* < .05). The cytotoxic effects of glyphosate were also evident, as observed by significant decrease in mitotic index (MI). The present results indicate that glyphosate is clastogenic and cytotoxic to mouse bone marrow.

## 1. Introduction

Pesticides, including herbicides, insecticides, and fungicides are used extensively to improve crop yields and as a result, they accumulate in the environment and humans unavoidably exposed to them [1]. Pesticides tend to be very reactive compounds that can form covalent bonds with various nucleophilic centers of cellular biomolecules, including DNA [2–4]. Because of their biological activity, the indiscriminate use of pesticides may cause undesired effects to human health. For instance, the induction of DNA damage can potentially lead to adverse reproductive outcomes, the induction of cancer, and many other chronic diseases [5–8]. Epidemiological studies demonstrated that occupational exposure to some pesticides may be related to several kinds of cancer, including leukemia [9], bladder [10], and pancreatic cancers [11].

To assess the genetic damage induced by physical and chemical agents including pesticides, various test systems have been described in bacteria, in mammalian cells in vivo and in vitro and in plants [12–14]. Arguably, the most reliable genotoxicity evaluation for human health risk is conducted in mammals by the induction of chromosomal aberrations (CAs) and micronuclei (MN). In this regard, particular attention is focused on CAs because these are considered as early warning signals for neoplastic development [15, 16]. MN are defined as small, round, DNA containing cytoplasmic bodies formed during cell division by loss of acentric chromatin fragments and/or whole chromosomes and are used as a fast and reliable assay for detecting clastogenic or aneugenic action [17]. CAs qualitatively and quantitatively detect clastogenic activity, while the MN assay detects both clastogenic effects and damage to the mitotic apparatus, some of which might have aneugenic consequences[18]. 

**Figure 1:**
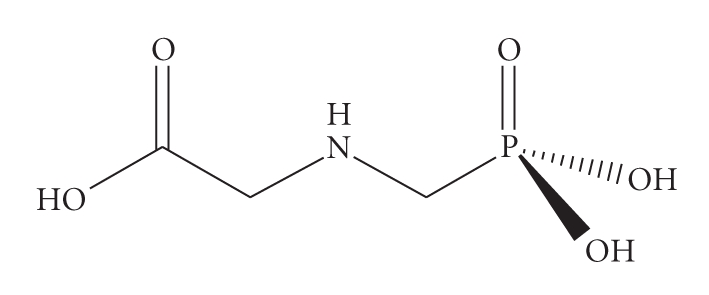
Chemical structure of glyphosate.

Glyphosate [chemical name: N-(phosphonomethyl)glycine - isopropylamine (IPA) salt; C_3_H_8_NO_5_P; Figure 1], commonly sold in the commercial formulation named Roundup, Rodeo, Touchdown, and so forth, has been a frequently used herbicide on both cropland and noncropland areas of the world since its introduction in the 1970s [19]. Roundup (CAS # 1071-83-6) is a liquid water soluble organophosphorus herbicide, containing glyphosate as its active ingredient and surfactant (polyoxyethyleneamine) that enhances the spreading of spray droplets when they contact foliage. As a herbicide Roundup works by being absorbed into the plant not only through its leaves but also through soft stalk tissue and applied at concentrations ranging from 0.26–1.152% of active ingredient, that is, glyphosate (20). Plants treated with glyphosate slowly die over a period of days or weeks [20]. Glyphosate is transported throughout the plant where it inhibits the shikimic acid pathway, which participates in the biosynthesis of phenylalanine and tyrosine and is also the major pathway in the biosynthesis of most plant phenolics [21]. Because this specific biologic pathway operates only in plants and microorganisms, the mechanism is not considered to be a risk for humans. Nevertheless, genotoxic, hormonal, and enzymatic effects of glyphosate in mammals have been reported [20, 22–25]. In rats, glyphosate was found to decrease the activity of some detoxifying enzymes, cytochrome P-450, and monooxygenase activities and the intestinal activity of aryl hydrocarbon hydroxylase when injected into the abdomen [26].

Li and Long [27] reported nonmutagenic effects from glyphosate in *Salmonella typhimurium*, *Escherichia coli*, *Bacillus subtilis*, Chinese hamster ovary cells gene mutation assay and chromosomal aberration in rat bone marrow cells. However, some other studies stated that glyphosate treatment on human lymphocytes in vitro resulted in increased sister chromatid exchanges [18, 22], CAs [22, 28], and oxidative stress measured by glucose 6-phosphate dehydrogenase (G6PD, marker of changes in the normal cell redox state) enzyme activity [22]. Roundup was associated with increased DNA adducts in mice [23] and DNA damage in *Rana catesbeiana* tadpolesas assessed by using Comet assay test [29]. Beside these, several assays also have demonstrated genotoxic activities of roundup, such as induction of reverse mutation in *S. typhimurium* (TA98 and TA100) and sex-linked recessive lethal mutation in *Drosophila melanogaster* [12, 28, 30] whereas glyphosate alone did not show these effects. In mammalian cells glyphosate was not also mutagenic [19]. Thus, so far there have been conflicting reports on the genotoxic hazards associated with the use of glyphosate.

On the basis of the information available, U.S. Environmental Protection Agency [31] and the World Health Organization [32] reviewed the toxicology data on glyphosate and concluded that glyphosate is not mutagenic or carcinogenic in humans. On the contrary, few recent studies have demonstrated cytotoxic effects of glyphosate [22, 33, 34]. Considering the widespread and frequent use of glyphosate throughout the world, ongoing risk assessment is of importance. In the present study we reported the genotoxic potential of glyphosate in mouse bone marrow cells.

## 2. Materials and Methods

### 2.1. Chemicals

Roundup containing active ingredient glyphosate >41% SL (IPA salt) was purchased from, Monsanto India Ltd. (Mumbai, India). Benzo(a)pyrene [B(a)P], colchicine and Giemsa were obtained from Sigma Chemical Company (St. Louis, USA). The rest of the chemicals used in the study were of analytical grade purity and obtained locally.

### 2.2. Animals and Treatment

Swiss albino mice (Male, 18 ± 2 g b.wt.; age: 10–12 weeks) were obtained from the Indian Institute of Toxicology Research (Lucknow, India) animal breeding colony. The ethical approval for the experiment was obtained from Institutional Ethical Committee. Animals were randomly selected and housed in polycarbonate boxes with steel wire tops and rice husk bedding. They were maintained in controlled atmosphere of 12 hours dark/light cycle, 25 ± 2°C temperature, and 57 ± 7% humidity with free access to pelleted feed (M/s. Ashirwad, Chandigarh, India) and fresh tap water.

The animals were divided into four groups of 15 animals each in two sets. The animals of group I were used as a control group and intraperitonialy (*i.p.*) treatment DMSO (0.2 mL, once only) was given. The animals of group II were served as positive control and only B(a)P was given at the single dose of 100 mg/kg b.wt. *i.p.* In groups III and IV single dose of glyphosate (diluted appropriately in DMSO) was given *i.p.* at the dose of 25 and 50 mg/kg b.wt., respectively.

### 2.3. Chromosomal Aberration Assay

After completion of the treatment period 5 animals from each group of set 1 were sacrificed at the sampling time of 24, 48, and 72 hours, respectively, by cervical dislocation (colchicine was given at a dose of 4 mg/kg of the b.wt. at 2 hours prior to sacrificing the animals to arrest cycling cells in metaphase). Cytogenetic analysis was performed as per the protocol of Preston et al. [35]. Briefly, the bone marrow was flushed out from both femurs using Hanks buffered salt solution (pH 7.2). The cells were centrifuged at 1000 rpm for 5 minutes and the pellet was redispersed in a hypotonic solution of 0.56% (w/v) KCl for 30 minutes at 37°C to permit osmotic swelling of cells. Swollen cells were fixed in ice-cold Carnoy's fluid, dropped onto slides, and stained with phosphate-buffered 5% Giemsa solution. A total of 75 well spread metaphase plates per animal in each group was analyzed for chromosomal aberrations at a magnification of 100x and the mitotic index (MI) was calculated from a scan of 2000 cells per animal. The chromosomal aberrations were classified as breaks, fragments, and exchanges. The incidence of aberrant cells was expressed as the percentage of damaged cells (aberrant metaphases).

*Mitotic Index (MI)* %:

(1) $$\frac{\text{Number of dividing cells}\times 100}{\text{Total number of bone marrow cells counted}},$$

*Incidence of aberrant cells* (%):

(2) $$\frac{\text{Total }\;\text{ number }\;\text{ of }\;\text{ aberrant }\;\text{ metaphases}\times 100}{\text{Total }\;\text{ number }\;\text{ of }\;\text{ metaphases }\;\text{ counted}}.$$

### 2.4. Micronuclei Induction Assay

The rest of 5 animals from each group of set 2 were sacrificed after 24, 48, and 72 hours of treatment and the frequency of micronucleated polychromatic erythrocytes (MNPCEs) was evaluated using a modified protocol of Schmid [36]. The bone marrow was flushed from both femurs using Hanks' buffered salt solution, 1% (w/v) bovine serum albumin, and 0.15% (w/v) EDTA (pH 7.2). Evenly spread bone marrow smears were stained by using the May-Grunwald and Giemsa protocol. A minimum of 2000 erythrocytes was scored for each treated and control group. The stained slides were scored for number of MNPCE's/1000 PCE's.

### 2.5. Statistical Analysis

The data was analyzed for mean values and standard error (mean ± SE) for all groups. Statistical comparisons were made using Students *t*-test, and *P* < .05 was considered significant.

## 3. Results

**Figure 2:**
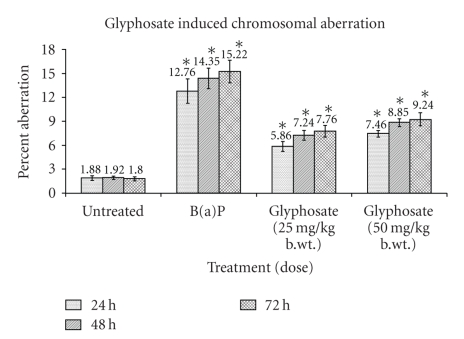
Mutagenic activity of glyphosate in Swiss albino mice showing incidence of aberrant cells at sampling time of 24, 48, and 72 hours. Values are expressed as mean ± SE of 5 animals. *Represent significant increase over untreated control group at their respective sampling time. Data are significant as *P* < .05.

In the results of chromosomal aberration assay, the percent incidence of aberrant cells in positive control B(a)P treated groups were found to be 12.76, 14.35, and 15.22 in 24, 48, and 72 hours of sampling time, respectively, in comparison to 1.88, 1.92, and 1.75 of untreated group I (Table 1, Figure 2). The frequency of percentage aberrant cells was also found to be significantly (*P* < .05) increased in glyphosate treated groups in dose- and time-dependent manner. The frequency of percent aberrant cells in glyphosate (25 mg/kg b.wt.) treated group III was found increased to 5.86, 7.24, and 7.76 in 24, 48, and 72 hours of sampling time, respectively, while in group IV (50 mg/kg b.wt.) it was 7.46, 8.85, and 9.24, respectively (Table 1, Figure 2).

**Figure 3:**
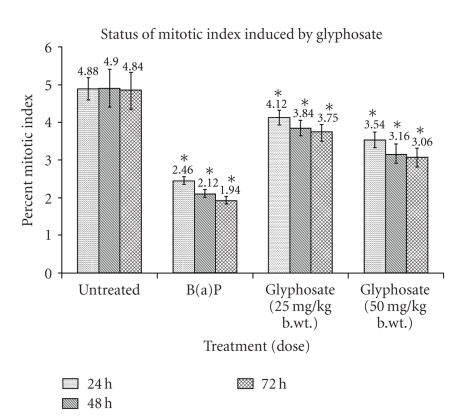
Cytotoxic effects glyphosate in Swiss albino mice indicated by decrease in mitotic index (MI) at 24, 48, and 72 hours of sampling time. Values are expressed as mean ± SE of 5 animals. *Represent significant decrease over untreated control group at their respective sampling time. Data are significant as *P* < .05.

Significant decrease in MI after B(a)P treatment was noticed and evaluated as percentage of dividing cells which was found to be 2.46, 2.12, and 1.94 in group II in comparison to 4.88, 4.90, and 4.84 of untreated control group I (Table 2, Figure 2). A significant (*P* < .05) decrease in MI was also observed in glyphosate treated groups III and IV in comparison to untreated controls (group I). Low-dose (25 mg/kg b.wt) glyphosate resulted in significant decrease in MI by 4.12, 3.84, and 3.75 in 24, 48, and 72 hours of treatment while high dose (50 mg/kg b.wt.) resulted in 3.54, 3.16, and 3.06, respectively (Table 2, Figure 3).

**Figure 4:**
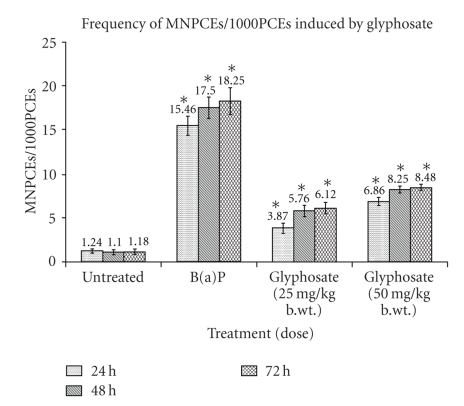
Mutagenic activity of glyphosate in Swiss albino mice showing increased micronuclei (MN) induction at sampling time of 24, 48, and 72 hours. Values are expressed as mean ± SE of five animals. *Represent significant increase over untreated control group at their respective sampling time. Data were significant as *P* < .05. MNPCEs: Micronucleated polychromatic erythrocytes and PCEs: polychromatic erythrocytes.

The frequency of MNPCEs/1000PCEs in the present study was 15.46, 17.50, and 18.25 in 24, 48, and 72 hours of B(a)P treatment (group II) and which was 1.24, 1.10, and 1.18 in control group I (Table 2, Figure 3). Glyphosate (25 mg/kg b.wt.) induced micronuclei induction in group III was 3.87, 5.76, and 6.12 whereas in group IV (50 mg/kg b.wt. glyphosate treated animals) it was 6.86, 8.25, and 8.48, in 24, 48, and 72 hours of sampling period, respectively, (Table 2, Figure 4), suggesting the genotoxic potential of glyphosate.

## 4. Discussion

Results of the present study reveals that single dose of glyphosate caused significant incidence of chromosomal aberration and induction of micronuclei in a dose- and time-dependent manner. Various cytogenetic results on commercial glyphosate are problematic. They may depend on purity of the active agent and on the nature of inert components. Surfactants and other inert compounds were previously suggested to increase the toxicity of the herbicide [37]. In a recent study, *Caiman latirostris* embryos were exposed at early embryonic stage to different sublethal concentrations of Roundup (range from 50–1750 *μ*g/egg), results from both the comet assay and the MN test revealed a concentration dependent effect [4].

Glyphosate reported for positive clastogenic and genotoxic effects in vitro [22, 27] which are consistent with our results (Tables 1 and 2). Chromosomal damage is considered to detect early effects of xenobiotic insult and evaluation of the frequency of CAs is a sensitive cytogenetic assay for detecting exposure to mutagens and carcinogens [15]. In the present study, glyphosate induced CAs could be attributed to early changes either an increase in induced DNA lesions or interference with their repair (Table 1, Figure 1). Glyphosate has been reported to cause DNA damage in erythrocytes of bullfrog tadpoles (*R. catesbeiana*) [29]. However, few studies reported that glyphosate is weak or nonclastogenic in vivo [18, 28, 38]. 

The MN induction assay was used as an additional sensitive biological indicator of the damage to somatic cell genome of subjects exposed to pesticide mixtures occupationally. It is known that the appearance of MN is related to the loss of chromosome fragments due to chromosome breaks [39]. Our results revealed that there was elevation in the number of micronuclei in the glyphosate exposed animals. Because MN could be the consequence of the mitotic spindle malfunction, it is possible that the glyphosate could also express an aneugenic mode of action as inhibiting cell division and mitotic spindle apparatus.

The molecular mechanisms responsible for the genotoxicity of glyphosate are not yet known clearly. However, the CAs and the micronucleus formation observed in animals clearly indicate that these compounds interact with chromatin DNA and induce damage there. Such interactions/DNA damage may be caused by an increased incidence of alkali labile sites in DNA as observed in kidney and liver with glyphosate treatment in CD-1 mice [23]. Alkali labile sites are generally produced at abasic sites in DNA and may be revealed under conditions that denature DNA secondary structure. Peluso et al. [23] also reported a dramatic increase in the number of oxidized guanine, 8-hydroxylguanine (8-OHdG), residues in DNA of liver cells from mice treated with glyphosate which also may be the reason of chromosomal damage in bone marrow cells of mice as observed in our study. It has also been shown in our study that CAs and MN induction increases in time as well as dose-dependent manner. It could be due to the glyphosate induced toxicity which produces reduced repair of spontaneous 8-OHdG and lead to an accumulation of oxidation products [23].

The sensitivities of two cytogenetic tests, chromosome analysis and the micronucleus test, were compared by using mice exposed to the substances glyphosate and B(a)P (Tables 1 and 2). Both test systems proved equally sensitive for genotoxicity assessment. Glyphosate at the tested doses significantly increased both the CAs rates and the MN induction in comparison to control. Thus, our results indicate that glyphosate is able to induce CAs and MN accompanied by inhibition of cell proliferation in Swiss albino mice following *i.p.* administration. In view of the earlier reports on mutagenic activity of glyphosate in laboratory experiments and from the present study, further studies are needed to assess the possible health hazard from glyphosate.

## Figures and Tables

**Table 1:** Effect of glyphosate treatment on induction of chromosomal aberration in swiss albino mice.

		Groups	Untreated	B(a)P	Glyphosate	Glyphosate
				(100 mg/kg b.wt)	(25 mg/kg b.wt)	(50 mg/kg b.wt)
		Breaks	0.36 ± 0.1	5.65 ± 0.4	2.86 ± 0.2	3.79 ± 0.16
		Fragments	0.17 ± 0.01	1.59 ± 0.03	0.39 ± 0.1	1.94 ± 0.02
	24 hours	Exchange	0.26 ± 0.02	0.69 ± 0.2	0.47 ± 0.3	0.41 ± 0.01
	of treatment	Multiple damage	1.02 ± 0.07	4.83 ± 0.3	2.14 ± 0.4	1.32 ± 0.07
		Total no. of	1.81 ± 0.03	12.76 ± 0.17*	5.86 ± 0.12*	7.46 ± 0.14*
		aberrant cells				
Number of		Breaks	0.33 ± 0.2	6.84 ± 0.5	3.37 ± 0.05	4.51 ± 0.07
aberrant cells		Fragments	0.19 ± 0.01	2.63 ± 0.7	0.46 ± 0.03	0.89 ± 0.01
(%) after	48 hours	Exchange	0.23 ± 0.1	0.83 ± 0.03	0.59 ± 0.01	0.54 ± 0.02
	of treatment	Multiple damage	1.17 ± 0.04	4.05 ± 0.04	2.82 ± 0.06	2.91 ± 0.16
		Total no. of	1.92 ± 0.03	14.35 ± 1.27*	7.24 ± 0.15*	8.85 ± 0.14*
		aberrant cells				
		Breaks	0.34 ± 0.02	6.91 ± 0.10	4.42 ± 0.07	4.49 ± 0.13
		Fragments	0.15 ± 0.01	2.93 ± 0.04	0.53 ± 0.02	0.82 ± 0.02
	72 hours	Exchange	0.19 ± 0.01	1.65 ± 0.06	0.47 ± 0.03	0.63 ± 0.02
	of treatment	Multiple damage	1.12 ± 0.04	3.73 ± 0.1	2.34 ± 0.09	3.30 ± 0.15
		Total no. of	1.80 ± .05	15.22* ± 1.19	7.76 ± 0.4*	9.24 ± 0.18*
		aberrant cells				

Mean ± SE of animals *n* = 5.

*P < .05.

**Table 2:** Effects of glyphosate treatment on mitotic index and micronuclei induction in swiss albino mice.

Groups (treatment)	Mitotic index (MI) after treatment			Micronuclei induction (MNPCEs/1000PCEs) after treatment		
	24 hours	48 hours	72 hours	24 hours	48 hours	72 hours
Group I	4.88 ± 0.06	4.90 ± 0.02	4.84 ± 0.04	1.24 ± 0.01	1.10 ± 0.01	1.18 ± 0.03
(untreated)						
Group II B(a)P	2.46 ± 0.09^#^	2.12 ± 0.01^#^	1.94 ± 0.02^#^	15.46 ± 0.03*	17.50 ± 0.10*	18.25 ± 0.12*
(100 mg/kg b.wt)						
Group III (glyphosate	4.12 ± .05^#^	3.84 ± 0.04^#^	3.75 ± 0.03^#^	3.87 ± 0.02*	5.76 ± 0.08*	6.12 ± 0.07*
dose 25 mg/kg b.wt)						
Group IV (glyphosate	3.54 ± 0.01^#^	3.16 ± 0.03^#^	3.06 ± 0.01^#^	6.86 ± 0.04*	8.25 ± 0.04*	8.48 ± 0.09*
dose 50 mg/kg b.wt)						

Data shows mean ± SE of 5 animals in each group.

^*#*^ *P* < .05 represents significant decrease as compared to untreated control.

*P < .05 represents significant increase as compared to untreated control.

MNPCEs: Micronucleated polychromatic erythrocytes;

PCEs: Polychromatic erythrocytes.
